# The dimerization domain of *Pf*CENP-C is required for its functions as a centromere protein in human malaria parasite *Plasmodium falciparum*

**DOI:** 10.1186/1475-2875-13-475

**Published:** 2014-12-04

**Authors:** Garima Verma, Namita Surolia

**Affiliations:** Molecular Parasitology Laboratory, Molecular Biology and Genetics Unit, Jawaharlal Nehru Centre For Advanced Scientific Research, Jakkur, Bangalore, 560064 India

**Keywords:** *Plasmodium falciparum*, *Pf*CENP-C, Functional complementation, *Pf*CENP-C motif, *Pf*CENP-C dimerization domain

## Abstract

**Background:**

The conserved centromere-associated proteins, CENH3 (or CENP-A) and CENP-C are indispensable for the functional centromere-kinetochore assembly, chromosome segregation, cell cycle progression, and viability. The presence and functions of centromere proteins in *Plasmodium falciparum* are not well studied. Identification of *Pf*CENP-C, an inner kinetochore protein (the homologue of human CENP-C) and its co-localization with *Pf*CENH3 was recently reported. This study aims to decipher the functions of inner kinetochore protein, *Pf*CENP-C as a centromere protein in *P. falciparum*.

**Methods:**

Bio-informatic tools were employed to demarcate the two conserved domains of *Pf*CENP-C, and the functions of *Pf*CENP-C domains were demonstrated by functional complementation assays in the temperature sensitive (TS) mutant strains (mif2-3 and mif2-2) of *Saccharomyces cerevisiae* with MIF2p (the yeast homologue of CENP-C) loss-of-function. By site-directed mutagenesis, the key residues essential for *Pf*CENP-C functions were determined. The chromatin immunoprecipitation was carried out to determine the *in vivo* binding of *Pf*CENP-C to the *Plasmodium* centromeres and the *in vivo* interactions of *Pf*CENP-C with *Pf*CENH3, and mitotic spindles were shown by co-immunopreciptation experiments.

**Results:**

The studies demonstrate that the motif and the dimerization domain of *Pf*CENP-C is able to functionally complement MIF2p functions. The essential role of some of the key residues: F1993, F1996 and Y2069 within the *Pf*CENP-C dimerization domain in mediating its functions and maintenance of mitotic spindle integrity is evident from this study. The pull-down assays show the association of *Pf*CENP-C with *Pf*CENH3 and mitotic spindles. The ChIP-PCR experiments confirm *Pf*CENP-C-enriched *Plasmodium* centromeres. These studies thus provide an insight into the roles of this inner kinetochore protein and establish that the centromere proteins are evolutionary conserved in the parasite.

**Conclusions:**

*Pf*CENP-C is a true CENP-C homologue in *P. falciparum* which binds to the centromeric DNA and its dimerization domain is essential for its *in vivo* functions as a centromere protein. The identification and functional characterization of the *P. falciparum* centromeric proteins will provide mechanistic insights into some of the mitotic events that occur during the chromosome segregation in human malaria parasite, *P. falciparum*.

**Electronic supplementary material:**

The online version of this article (doi:10.1186/1475-2875-13-475) contains supplementary material, which is available to authorized users.

## Background

During cell division, the fidelity in chromosome segregation is ensured by the specialized locus on the chromosome, the centromere. The kinetochore proteins assemble on the centromeric chromatin and mediate the attachment of the microtubule spindles to the chromosomes. In all the eukaryotes studied so far, CENP-A and CENP-C together form the core components of the centromeric chromatin and are indispensable for centromere formation and function. CENP-C is an integral part of the inner kinetochore plate and serves as an interface between the centromeric chromatin and the outer kinetochore plate to which spindle microtubules attach [1–5]*.* A functional kinetochore assembly depends on the recruitment of the CENP-C to the CENP-A-containing centromeric nucleosomes and the subsequent CENP-C-dependent recruitment of a subset of inner and outer kinetochore proteins on the centromere [6–12]. Like CENP-A, CENP-C constitutively localizes to the active centromeres and is required for the kinetochore assembly and timely progression during cell cycle [1, 3, 13–20]. Loss of CENP-C function results in abolishing the centromere-kinetochore assembly, chromosome mis-segregation, metaphase/anaphase block and cell death, suggesting its indispensable role in proper chromosome segregation and viability in the eukaryotes [2, 4, 13, 18, 21–26].

Detailed analyses of CENP-C revealed that it is composed of different domains, each with specialized functions [6, 17, 27–29]. It comprises of an amino terminal domain, a central region, a signature CENP-C motif, and a carboxy terminal dimerization domain. The amino terminal domain of CENP-C is shown to confer oligomerization and instability on CENP-C and is not required for centromere targeting [27, 30]. This domain binds directly to the components of Mis12 kinetochore complex and prevents the excessive accumulation and mistargeting of CENP-C to the non-centromeric sites [4, 20, 27, 30, 31]. The central region of CENP-C has been shown to carry a DNA-binding domain with an overlapping centromere-targeting domain [3, 17, 27, 28, 32, 33]. The signature CENP-C motif binds to the CENP-A-containing centromeric nucleosomes, centromeric α-satellite DNA and is required for the centromere targeting [25, 34–37]. The dimerization domain is responsible for the higher order structural organization of CENP-C, self-dimerization, centromeric targeting and *in vivo* kinetochore assembly [6, 10, 27, 28, 30].

In *Saccharomyces cerevisiae*, the mutations in the MIF2p domains, namely, the CENP-C motif and the dimerization domain, lead to impaired centromere-kinetochore structure, metaphase to anaphase delay and chromosome mis-segregation [6, 25, 36, 37]. Thus, this mitotic fidelity gene is shown to have an essential role in chromosome segregation and mitotic spindle integrity and the cells deficient in MIF2p show abnormal phenotypes, defective spindle morphologies and delayed progression during mitosis [21, 36, 38].

Recent studies on the centromere-specific H3 variant have contributed to the current understanding of centromere proteins in *Plasmodium falciparum*[39]. The *Plasmodium* centromeres are enriched with *Pf*CENH3 and are important for centromere assembly and propagation [40]. The structural-functional determinants of *Pf*CENH3 that confer centromere specific functions and the association of *Pf*CENH3 with another putative inner kinetochore protein *Pf*CENP-C have also been demonstrated in this parasite [39].

This study reports the identification of various CENP-C domains and detailed functional dissection of its dimerization domain in *P. falciparum*. The two domains of *Pf*CENP-C, namely, the CENP-C motif and the dimerization domain are mapped using bio-informatic analysis and their roles as a centromere protein are tested by the functional complementation of MIF2p. In budding yeast, the temperature-sensitive (TS) mutants, mif2-2 and mif2-3 harbour different mutations which results in MIF2p loss-of–function. The mif2-2 TS strain carries a ‘S309L’ mutation in the CENP-C motif as well as a ‘S500F’ mutation in its dimerization domain [21, 34, 36], the mif2-3 TS strain has a ‘P505L’ mutation in the MIF2p dimerization domain (MIF2p^P505L^) [6, 34] that confers the TS phenotype. While, the *Pf*CENP-C dimerization domain could functionally substitute for the MIF2p loss-of-function in the mif2-3 TS mutant, it could partially complement the MIF2p loss-of-functions in mif2-2 TS mutants. The *Pf*CENP-C motif show partial rescue in the mif2-3 as well as mif2-2 TS mutants, respectively. These TS yeast cells at non-permissive temperature show aberrant phenotype features such as chromosome mis-segregation and broken microtubules that are rescued upon the expression of *Pf*CENP-C dimerization domain. The indispensable role of the *PfC*ENP-C dimerization domain in mediating the centromere protein functions is confirmed by the mutational analysis of the conserved residues (F1993, F1996 and Y2069) within the *PfC*ENP-C dimerization domain as compared to the conserved N2060 residue in the *Pf*CENP-C dimerization domain corresponding to the MIF2p N514 residue, which is not required for its functions. The *Pf*CENP-C^P2051L^ dimerization domain mutant corresponding to MIF2p^P505L^ TS mutation, as present in the mif2-3 TS mutant strain also fails to overcome the growth defects. Severe functional and phenotypic defects on growth, chromosome segregation and mitotic spindle structures are observed upon the expression of *PfC*ENP-C dimerization domain mutants, demonstrating the importance of a functional dimerization domain in the kinetochore assembly, chromosome segregation and maintenance of the mitotic spindle integrity.

In addition, ChIP-PCR data shows the enrichment of *Pf*CENH3 and *Pf*CENP-C on the centromeric regions of *P. falciparum* as compared to the non-centromeric regions. The immunoprecipitation experiments demonstrate the interactions of *Pf*CENP-C with *Pf*CENH3 and tubulin. Thus, the data presented here establishes the putative *Pf*CENP-C as a true CENP-C homologue and the role of its dimerization domain in mediating the functions of *Pf*CENP-C.

## Methods

### *Plasmodium falciparum*culture

The *P. falciparum* FCK2 (a local strain of Karnataka, India) was cultured in O^+^ human erythrocytes, RPMI 1640 medium supplemented with 25 mM HEPES, 0.2% NaHCO_3_ and 10% (v/v) heat-inactivated O^+^ human serum by standard candle jar method [41]. Tight synchronization of cultures was performed by 5% D-sorbitol [42] and the parasites were isolated by 0.15% (w/v) saponin lysis followed by subsequent washes of the parasite pellet with cold phosphate-buffered saline pH7.4 (1× PBS).

### Bio-informatics analysis

Using the BLASTp [43], the amino acid sequences of conserved CENP-C motif and the C-terminus dimerization domain of human CENP-C were used as queries to blast against the whole *P. falciparum* genome database [44]. Using ClustalW and Espript 3.0, the multiple sequence alignments were performed between *Pf*CENP-C and its homologues. Percent pair-wise similarities between the full-length CENP-C homologues were determined by Needleman-Wunsch alignment.

### Antibodies

The peptide sequence N- VEVILVEKKLKKKKQKC-C was used to generate *Pf*CENP-C polyclonal antibodies in mice followed by affinity purification with the respective peptide (Genemed Synthesis, USA). To stain the mitotic spindles, mouse monoclonal anti α-tubulin (Calbiochem, USA) was used. Monoclonal anti-GFP (Roche, USA) was used for Western blot and immunofluorescence assays.

### Western blot analysis

The isolated parasites from various asexual stages were lysed in RIPA buffer (150 mM NaCl, 50 mM Tris-HCl, pH 7.4, 1% NP-40, 0.25% sodium deoxycholate, 1 mM EDTA, 1 mM sodium orthovanadate, protease inhibitor cocktail) [45] and mechanically sheared by passing through 1-ml syringe. The standard Bradford method [46] was used to estimate the protein concentration of the lysates. The whole cell parasite extract was resolved on 6 and 12% SDS polyacrylamide gel electrophoresis (Mini Protean Tetrad apparatus, Biorad, USA) followed by immunoblotting on PVDF membrane (Millipore, India). The parasite extracts were probed using anti-*Pf*CENP-C (1:1,000), anti-GAPDH (1:2,000, Sigma-Aldrich, Germany), anti-*Pf*CENH3 (1:2,000, Genemed Synthesis, USA) and anti-α tubulin (1:2,000, Calbiochem, USA) followed by incubation with goat anti-mouse and anti-rabbit secondary antibodies conjugated with horseradish peroxidase (1:3,000, Bangalore Genei, India). ECL substrate (Biorad, USA) was used to detect the signals of the Western analyses.

In *S. cerevisiae*, the Western blot analyses were carried out by growing the yeast strains expressing different constructs both at permissive and restrictive temperatures at A_600_ = ~1. For lysate preparation, the yeast pellets were washed twice with autoclaved H_2_0 and lysed in 50% TCA for 1 hr at -80°C followed by two washes with 80% acetone. The air-dried pellets were resuspended in 0.1% SDS/0.1 N NaOH. Western blot analyses were carried out on the lysates and the GFP-tagged *Pf*CENP-C domains were detected by mouse monoclonal anti-GFP (1:3,000, Roche, USA).

### Yeast strains, expression constructs and site-directed mutagenesis

For the functional complementation assays, two different TS strains deficient in MIF2p, Y06477 (mif2-3: KanR: his3^1 leu2^0 ura3^0 met15^0); 6848-4-2 (Mat a ura3 mif2-2) [21, 34, 36], and the wild type (WT) strain DMA3865 (Mat a; ura10^Kan his3^1 leu2^0 ura3^0 met15^0) were used. YPDU-agar and YPDU broth (1% yeast extract, 2% peptone, 2% dextrose and 0.01% uracil) were used to grow all the strains at 23°C (permissive temperature). The mif2-2 TS mutant strain has ‘S309L’ mutation in the CENP-C motif and a ‘S500F’ mutation in its dimerization domain [21, 34, 36]. The mif2-3 TS mutant strain has a ‘P505L’ point mutation in the MIF2p dimerization domain [6, 34]. Both the TS mutant strains show growth arrest at 37°C (non-permissive temperature). The yeast expression vector p415ADH-containing ADH1 promoter, leucine selectable marker and the terminator sequences was used for cloning the various *Pf*CENP-C domains between the XbaI and BamHI restriction enzyme sites. To clone the N-terminal region (NTD) of *Pf*CENP-C, first 540 nucleotides of full-length *Pf*CENP-C was used. Based on the sequence comparisons, the nucleotide sequences spanning the *Pf*CENP-C motif and the *Pf*CENP-C dimerization domain were used for cloning these respective *Pf*CENP-C domains in p415ADH. The full-length MIF2p from *S. cerevisiae* was also cloned into the XbaI and HindIII sites of p415ADH. The vector carrying *Pf*CENP-C domains, full-length MIF2p and empty vector alone were transformed into the WT strain (DMA3865) and the TS mif2-3 and mif2-2 mutant yeast cells (Y06477 and 6848-4-2) by lithium acetate method [47]. Synthetic media devoid of leucine was used to select the transformants. The Quick Change II XL site-directed mutagenesis kit (Stratagene, USA) was used to substitute the conserved amino acid residues in the *Pf*CENP-C dimerization domain following the protocol recommended by the manufacturer. The point mutations were incorporated by using the primers spanning the nucleotide coding for leucine and alanine for substitution of proline at 2051 position and F1993, F1996, N2060 and Y2069, respectively in the *Pf*CENP-C dimerization domain. The expression construct containing the 0RF (*Pf*CENP-C dimerization domain) was used as a template (10-50 ng) and amplified with the high fidelity *Pfu* polymerase. The methylated template DNA was digested by Dpn I for 1 hr at 37°C followed by transformation into DH5α competent cells. The desired mutations were confirmed by sequencing and transformed into the mif2-3 and mif2-2 TS mutants (Y06477 and 6848-4-2) and the WT strain (DMA3865).

### Functional complementation assay

To assess the complementation ability of *Pf*CENP-C domains, the mif2-3 and mif2-2 transformants expressing *Pf*CENP-C N-terminal region (NTD), *Pf*CENP-C motif, *Pf*CENP-C dimerization domain (DD), MIF2p and empty vector were streaked on the plates containing selective media devoid of leucine and fully supplemented YPDU agar, incubated at 23°C and 37°C for three days and imaged. For ten-fold spot dilution experiments, the yeast strains expressing the abovementioned expression constructs were grown in YPDU broth at 23°C to attain A_600_ = ~1. Serial dilutions starting from 5×10^5^ cells ml^−1^ to 5 × 10^−1^ cells ml^−1^ were prepared and spotted on YPDU plates and allowed to grow at 23°C and 37°C for three days. The mif2-3 and mif2-2 TS mutant cells expressing full-length MIF2p from *S. cerevisiae* and the empty vector alone (p415ADH) were used as positive and negative controls, respectively. The phenotypes of the various transformants were visualized and imaged by fixing the cells with 70% alcohol and Hoechst staining of the nuclei followed by confocal imaging. The gain-or loss-of-phenotypes was analysed and scored by imaging the bud morphologies and the nuclear staining.

### Indirect immunofluorescence assay

The tightly synchronized parasite cultures were processed for indirect immunofluorescence assays as previously described [48]. The parasites were washed with PBS and fixed using 4% paraformaldehyde and 0.0075% glutaraldehyde in PBS (EM grade, Polysciences Inc, USA) 30 min at room temperature and for permeabilization 0.05% Triton X-100 was used for 2 min at room temperature followed by blocking with 3% BSA for 1 hr at 4°C. Both the primary and secondary antibodies were diluted in 3% BSA and incubated for 1 hr at room temperature. The primary antibody, mouse anti-*Pf*CENP-C and the secondary antibody, Alexa Fluor 568 conjugated goat anti-mouse were used at 1:100 and 1:200 (Molecular Probes, USA) dilutions, respectively. Hoechst3328 (Sigma-Aldrich, USA) was used to stain nucleus followed by mounting over the glass slides using Vectashield (Sigma-Aldrich, USA).

In *S. cerevisiae*, the indirect immunofluorescence assays were performed on the mif2-3 and mif2-2 TS mutant cells expressing various *Pf*CENP-C domains, MIF2p and empty vector using protocol as previously described [47]. The cells were allowed to grow at A_600_ = ~1 and fixed with 37% formaldehyde (1/10^th^ culture volume) for 1 hr. Subsequently, the cells were washed with 0.1 M phosphate buffer (pH 6.4) and allowed to sphaeroplast using lyticase (Sigma-Aldrich, USA) and 2-mercapto ethanol at 30°C, 65 rpm. Teflon-coated slides incubated with poly-L-lysine (1 mg ml^−1^, Sigma-Aldrich, Germany) were used to adhere the cells. The cells on the slides were fixed in ice-cold methanol for 6 min at -20°C and ice-cold acetone for 30 sec at -20°C. The blocking was performed in a humid chamber with 2% skimmed milk in PBS for 30 min followed by incubation with anti-α tubulin (1:200) and anti-GFP (1:400) for 1 hr. The cells were then incubated with Alexafluor 488 secondary antibodies (1:200) for 1 hr. PBS washes were carried out between the two antibodies incubation and the nuclei were stained with Hoechst 3328 (Sigma-Aldrich, USA). All the confocal images were evenly adjusted for brightness and contrast.

### Chromatin immunoprecipitation (ChIP)

The ChIP assays were performed as described by [49, 50]. The parasite cultures were crosslinked by addition of 37% formaldehyde (Sigma-Aldrich, USA) to get a final concentration of 1% for 5 min at 37°C. The crosslinking was stopped by the addition of 0.125 M glycine for 5 min on ice. The crosslinked parasites were washed thrice in ice-cold PBS and 0.06% saponin was used for RBC lysis. After saponin lysis, the parasite pellet was again washed twice with 1× PBS (pH 7.4). The isolated crosslinked parasites were resuspended in cold lysis buffer (10 mM KCl, 10 mM Hepes pH 7.9, 0.1 mM EGTA pH 8.0, 0.1 mM EDTA pH 8.0, 1 mM DTT and protease inhibitor cocktail) and transferred to a douncer homogenizer for lysis with the addition of NonidetP-40 at a final concentration of 0.25%. The lysate was centrifuged at 16,000 × g for 10 min at 4°C. The pellet obtained was resuspended in SDS lysis buffer (50 mM Tris-HCl pH 8.1, 1% SDS and 10 mM EDTA). To get sheared chromatin, sonication was performed at high power with 30 sec on and offcyclings for 10 min (Diagenode Bioruptor). The sheared chromatin was collected by centrifugation at 12,500× g for 10 min at 4°C and diluted ten-fold in ChIP dilution buffer (1.1% Triton X-100, 0.01% SDS, 16.7 mM Tris-HCl pH 8.1, 150 mM NaCl and 1.2 mM EDTA). For chromatin-IP assays, the chromatin solutions were precleared with protein A or G agarose slurry (GE Healthcare, Sweden) and 50 μl mL^−1^ of salmon sperm DNA (Sigma-Aldrich, USA) for 2 hr at 4°C. The precleared chromatin solutions were incubated with *Pf*CENP-C and *Pf*CENH3 antibodies for 12 hr at 4°C. The immune complexes were collected by centrifugation at 1,000× g for 1 min and washed with three different wash buffers at 4°C, the low salt wash buffer (20 mM Tris-HCl pH 8.1, 2 mM EDTA, 0.1% SDS, 150 mM NaCl and 1% Triton X-100) followed by the high salt wash buffer (20 mM Tris-HCl pH 8.1, 2 mM EDTA, 0.1% SDS, 500 mM NaCl and 1% Triton X-100) and LiCl wash buffer (10 mM Tris-HCl pH 8.1, 0.25 M LiCl, 1 mM EDTA, 1% NP-40 and 1% deoxycholate). The final washes were performed with TE (10 mM Tris-HCl pH 8.0 and 1 mM EDTA, pH 8.0) buffer at room temperature. The elution was performed with freshly prepared elution buffer (0.1 M NaHCO3 and 1% SDS) at room temperature. To obtain DNA, the eluates and the input samples were de-crosslinked by incubating at 65°C followed by treatment with Proteinase K and RNaseA. The DNA was purified by phenol/chloroform/isoamyl alcohol extraction method. The immunoprecipitated as well as the input DNA samples were quantified and used as templates for semi-quantitative PCR reactions. To ensure that the amplifications are in linear range, the sub-saturation point was standardized to avoid end point analysis using Q-PCR amplification curves. For each set of primers, 5 cycles less than the point of saturation were performed and then the products were run on the gel for quantification. The fold enrichment (ChIP over input) for each centromeric as well as non-centromeric region was determined by quantifying the gel band intensities using AlphaImager.

### Immunoprecipitation

The immunoprecipitation assays were performed using *Pf*CENP-C and *Pf*CENH3 antibodies as previously described by [45]. The parasites were isolated at late trophozite and schizont stages and lysed in modified RIPA buffer (150 mM NaCl, 50 mM Tris-HCl, pH 7.4, 1% NP-40, 0.25% sodium deoxycholate, 1 mM EDTA, 1 mM sodium orthovanadate, protease inhibitor cocktail). Prior to IP, the lysates were precleared by incubating with Protein A or G agarose beads (GE Healthcare, Sweden) for 4 hr at 4°C and the antibodies were conjugated with the agarose beads. The protein concentration was estimated by standard Bradford method [46] based on which the amount of antibody/IP was determined (~10 ug of Ab for 5 ug of total protein). To immunoprecipitate the protein of interest, the total protein was incubated with the antibody-conjugated beads overnight at 4°C. The immunocomplexes were collected by centrifugation followed by three washes with chilled lysis buffer. The immunoprecipitated protein was eluted by 0.1 M glycine pH 3.4 followed by the neutralization. The eluates were subjected to Western blot analyses as described above.

## Results

### *Pf*CENP-C has conserved CENP-C motif and dimerization domain

**Figure 1:**
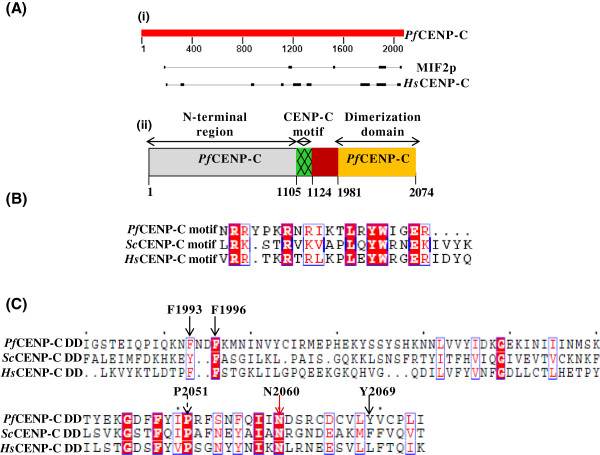
**Identification of** ***Pf*** **CENP-C and mapping of** ***Pf*** **CENP-C conserved domains, the CENP-C motif and dimerization domain. (A)** (i) The schematic representation of the regions of homology (black bars) between the full-length *Pf*CENP-C, MIF2p (*S. cerevisiae* CENP-C) and human CENP-C using BLASTp analysis. The regions of maximum homology are confined to the centre and the C-terminus region. Percent pair-wise similarity of full-length *Pf*CENP-C with full-length yeast MIF2p and human CENP-C were acquired by Needleman-Wunsch alignment. The full-length *Pf*CENP-C shares 10.8 and 17.5% amino acid sequence similarities with MIF2p and CENP-C, respectively. (ii) The schematic representation of the *Pf*CENP-C motif (green) and *Pf*CENP-C dimerization domain (orange) based on the sequence comparisons and *in silico* analyses. The region before the *Pf*CENP-C motif is represented as the N-terminal region (grey). The numbers indicate the amino acid positions. **(B)** Sequence comparison between the conserved CENP-C motifs. The CENP-C motif of *P. falciparum* shares 30 and 63% homology with *Sc*CENP-C and *Hs*CENP-C motifs, respectively. The red-shaded boxes represent the identical amino acids. **(C)** Sequence comparison of the dimerization domains (DD). The *Pf*CENP-C-DD shows 50 and 46% homology with the yeast and human CENP-C dimerization domains, respectively. The conserved amino acid residues in the dimerization domains of *Pf*CENP-C and MIF2p are indicated by arrows. (*Pf*- *Plasmodium falciparum*; Sc- *Saccharomyces cerevisiae*; *Hs*- *Homo sapiens*)*.*

The bio-informatic analyses have shown that CENP-C homologues display maximum sequence conservation at the CENP-C signature motif and the dimerization domain [29, 34, 51]. To identify CENP-C homologue in *P. falciparum,* protein sequences of the two conserved domains of human CENP-C, namely, the CENP-C signature motif and dimerization domain were used as queries for the BLAST analyses. The sequence comparisons revealed a putative CENP-C homologue [*PF*3D7_1021800] in th*e Plasmodium* database [44]. The BLASTp between *Pf*CENP-C, MIF2p and CENP-C shows the maximum sequence homology in the conserved regions of the CENP-C motif and the C-terminal dimerization domain (Figure 1A,i) and thus these conserved domains were mapped in *Pf*CENP-C (Figure 1A,ii). Percent pair-wise similarity of the full-length *Pf*CENP-C with full-length MIF2p and CENP-C were acquired by Needleman-Wunsch alignment. The full-length *Pf*CENP-C shares 10.8 and 17.5% amino acid sequence similarities with MIF2p and CENP-C, respectively. The *Pf*CENP-C-motif (amino acids 1105-1124) displays 30 and 63% amino acid sequence similarities with *S. cerevisiae* and human CENP-C motif, respectively (Figure 1B). The *Pf*CENP-C dimerization domain (amino acids 1981-2074) shares 50 and 46% amino acid sequence similarities with *S. cerevisiae* and human dimerization domains, respectively (Figure 1C). However, the amino terminal region of *Pf*CENP-C exhibit low sequence similarity with the *S. cerevisiae* or human CENP-C. The *in silico* analysis thus suggested the presence of a putative CENP-C homologue in *P. falciparum*.

### *Pf*CENP-C is constitutively expressed during all the intra-erythrocytic stages and localizes to the nuclear periphery

**Figure 2:**
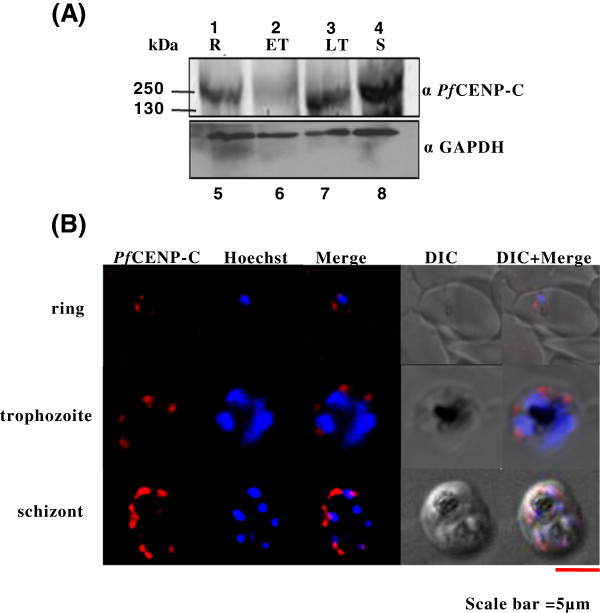
***Pf*** **CENP-C exhibits constitutive differential expression throughout the intra-erythrocytic stages and localize to the nuclear periphery. (A)** Stage-specific Western blot analyses of *Pf*CENP-C on the whole cell parasite lysates from tightly synchronized parasites at ring (R), early trophozoite (ET), late trophozoite (LT) and schizont (S) stages were probed with anti-*Pf*CENP-C (lanes 1-4) and anti-GAPDH (lanes 5-8) antibodies, respectively. **(B)** The confocal images for immunofluorescence assays representing the subcellular localization pattern of *Pf*CENP-C (red) and the Hoechst stained nucleus (blue) at ring, trophozoite and schizont stages of *P. falciparum,* respectively. Scale bar = 5 μm.

To determine the presence and the expression pattern of *Pf*CENP-C at the various intra-erythrocytic (IE) stages of the parasite, Western blot analysis was performed using anti-*Pf*CENP-C antibodies on the parasite lysates prepared from the tightly synchronized ring, early trophozoite, late trophozoite and schizont stages (Figure 2A, lanes 1-4). The presence of *Pf*CENP-C (244.9 kDa) is detected at all the stages but the expression is down regulated during the early trophozoite stage as compared to ring, late trophozoite and schizont stages, suggesting its differential constitutive expression at all the developmental stages of the IE cycle. The equal protein loading was confirmed by anti-GAPDH antibodies (Figure 2A, lanes 5-8). The sub-cellular localization of *Pf*CENP-C at various IE stages by indirect immunofluorescence assays using anti-*Pf*CENP-C antibodies was performed. The immunofluorescence assays show that *Pf*CENP-C localizes to the nuclear periphery at all the IE stages (Figure 2B). The *Pf*CENP-C has also been found to co-localize with *Pf*CENH3 at the nuclear periphery [39] which is consistent with the fact that CENP-C co-localizes with CENP-A throughout the cell cycle in other organisms like *Drosophila* and humans [20, 52]. These results thus confirm the presence of endogenous *Pf*CENP-C in the malaria parasite.

### The motif and the dimerization domain of *Pf*CENP-C functionally complements the MIF2p loss-of-function

The CENP-C specifically recognizes CENP-A in the centromeric nucleosome and promotes the functional centromere-kinetochore assembly [7–9, 12]. Mutations in the CENP-C motif and the dimerization domain lead to arrested growth, chromosome mis-segregation and mitotic delay [6, 25, 34, 36]. In order to determine whether the putative *Pf*CENP-C is a true homologue of CENP-C, the ability of the *Pf*CENP-C domains, namely, the N-terminal region, *Pf*CENP-C motif and *Pf*CENP-C dimerization domain to functionally complement MIF2p depleted TS mutant strains, mif2-3 and mif2-2 [34, 36] defective in mitotic chromosome segregation were tested.

**Figure 3:**
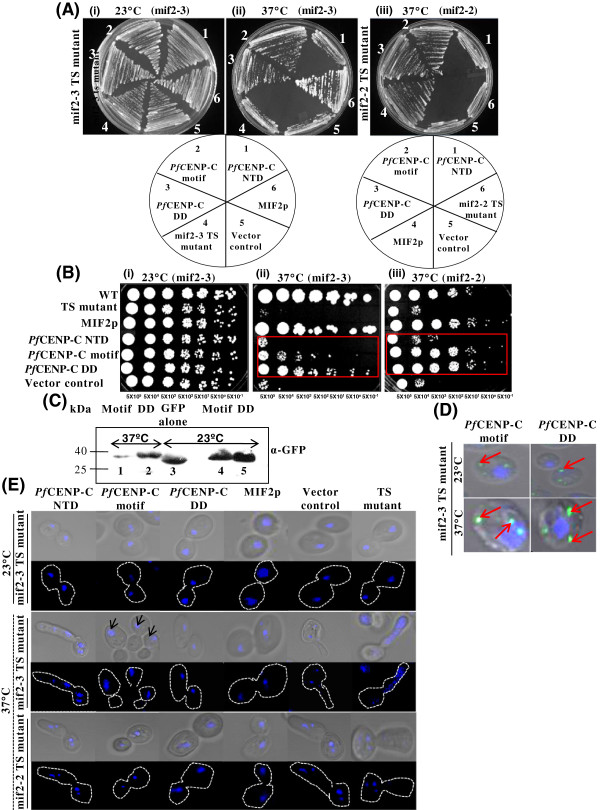
**Functional complementation of** ***Saccharomyces cerevisiae*** **MIF2p by the** ***Pf*** **CENP-C motif and** ***Pf*** **CENP-C dimerization domain.** (**A** and **B**, i-iii) Growth and ten-fold serial dilution assays with mif2-3 (i and ii) and mif2-2 (iii) TS mutants expressing MIF2p, empty vector, various *Pf*CENP-C domains: NTD, motif, and DD and the parent TS strains at 23 and 37°C, respectively. (i) At 23°C, the mif2-3 TS expressing abovementioned constructs display normal growth. (ii and iii) At 37°C, TS mutants, expressing *Pf*CENP-C-NTD, vector alone and the parent TS cells alone fail to grow, while MIF2p shows complete growth restoration. The mif2-3 TS mutants expressing *Pf*CENP-C motif and DD show partial and complete growth rescue, respectively while in mif2-2 TS cells, both *Pf*CENP-C motif as well as DD show partial growth rescue. The red boxes highlight the comparative growth of various *Pf*CENP-C domains at 37°C. **(C)** Western blot analysis showing the expression of GFP tagged *Pf*CENP-C motif (~29.68 kDa) and DD (~38.16 kDa) in mif2-3 TS mutants at 23 (lanes 4 and 5) and 37°C (lanes 1 and 2). **(D)** Confocal images of GFP tagged *Pf*CENP-C motif and the DD (green) showing their mislocalization (red arrows) to the non-centromeric sites both at 23 and 37°C. **(E)** Confocal images represent the phenotypes of mif2-3 and mif2-2 TS mutants carrying various constructs at 23 and 37°C, respectively. At 23°C, all the transformants show normal bud morphology with equal nuclear (blue) segregation. At 37°C, like parent TS mutants, *Pf*CENP-C-NTD and empty vector show aberrant bud morphology with fragmented nuclei. The *Pf*CENP-C motif expressing mif2-3 and mif2-2 TS mutants shows chromosome mis-segregation (black arrows) and proper chromosome segregation, respectively. However, the *Pf*CENP-C-DD expression in both the TS mutants restores the normal chromosome segregation. MIF2p expressing cells also exhibit normal phenotypes at 37°C. N-terminal region-NTD, dimerization domain-DD.

Functional complementation assays with WT strain, mif2-3 and mif2-2 TS mutant yeast cells expressing *Pf*CENP-C N-terminal region (NTD), *Pf*CENP-C motif, *Pf*CENP-C dimerization domain (DD), MIF2p, and an empty vector alone were performed at permissive (23°C) and non-permissive (37°C) temperatures (Figure 3A and 3B, i-iii ; see Additional file 1A and B). At the permissive temperature, all the mif2-3 and mif2-2 TS mutants expressing the abovementioned constructs show normal growth (Figure 3A,i; see Additional file 1A). At the non-permissive temperature (Figure 3A,ii and iii), the untransformed mif2-3 and mif2-2 TS mutants show severely retarded growth. The mif2-3 TS mutants carrying the *Pf*CENP-C motif and the *Pf*CENP-C dimerization domain show partial and complete growth restoration, respectively, the mif2-2 TS mutants expressing *Pf*CENP-C motif and the *Pf*CENP-C dimerization domain individually show only partial growth rescue. As compared to the complete growth rescue in the mif2-3 TS mutants, the *Pf*CENP-C dimerization domain partially rescues the growth defects in the mif2-2 TS mutants. Both the TS mutants devoid of the C-terminus region and expressing only the N-terminal region of *Pf*CENP-C show arrested growth at 37°C and fail to rescue the growth defects. The mif2-3 and mif2-2 TS mutants carrying the empty vector alone too, fail to grow at 37°C. As expected, both the TS mutants expressing MIF2p show complete rescue in the growth at the non-permissive temperature. Ten-fold serial dilution assays (Figure 3B, i-iii; see Additional file 1B) for monitoring the viability in mif2-3 and mif2-2 TS mutant cells also corroborates the complementation ability of *Pf*CENP-C motif and *Pf*CENP-C dimerization domain in MIF2p-depleted cells, establishing that both motif and the dimerization domain of *Pf*CENP-C are able to overcome the growth defects in its yeast counterpart but the *Pf*CENP-C dimerization domain appears to exhibit better functional complementation ability as compared to the *Pf*CENP-C motif.

To confirm the expression of the *Pf*CENP-C motif and the *Pf*CENP-C dimerization domain in the mif2-3 TS mutants, both the domains were tagged with GFP and Western blot analysis using anti-GFP antibodies were performed. The *Pf*CENP-C motif and the *Pf*CENP-C dimerization domain (DD) are expressed in the TS mutants at both the permissive as well as non-permissive temperatures (Figure 3C, lanes 1, 2, 4, and 5). The expression of CENP-C above the physiological level results in its mistargeting to the non-centromeric regions of the chromatin [24, 53]. To check whether *Pf*CENP-C motif and the *Pf*CENP-C dimerization domain localize to the yeast centromeres, indirect immunofluorescence assays with anti-GFP antibodies were performed. Owing to the overexpression of *Pf*CENP-C motif and the *Pf*CENP-C dimerization domain, multiple discrete spots over the nuclei are observed indicating the mislocalization of both the domains (Figure 3D). The N-terminal of CENP-C has been shown to destabilize the excess CENP-C, which prevents the CENP-C accumulation to the non-centromeric sites and ensures its correct centromeric targeting [30]. Extrapolating these results for *P. falciparum*, the mislocalization of the signals in the mif2-3 TS mutants can be attributed to the absence of the N-terminal region in the *Pf*CENP-C motif and the *Pf*CENP-C dimerization domain.

In *S. cerevisiae*, the deletion of the dimerization domain of MIF2p results in cells with TS phenotypes like abnormal large bud morphology, mitotic arrest and chromosome mis-segregation [6]. The large bud morphology is typically exhibited by cells arrested in S, G2 or M phases of the cell cycle and prolonged exposure to the non-permissive temperature leads to loss in viability [21]. To determine whether such phenotypic defects are rescued upon complementation of MIF2p by various *Pf*CENP-C domains, the phenotypes of the mif2-3 and mif2-2 TS mutant cells deficient for MIF2p function as compared to the TS mutant cells expressing different *Pf*CENP-C domains deletants, namely, the N- terminal region, CENP-C motif and the dimerization domain individually were examined and scored (Figures 3E, see Additional file 1C, i-iii). At 23°C, normal bud morphology and proper chromosome segregation is observed in both the TS mutants carrying the different *Pf*CENP-C deletion mutants. At 37°C, the majority of the parent TS mutant cells (mif2-3 and mif2-2) and the vector control show aberrant large bud morphology with dispersed and/or fragmented nuclei along with unviable cells. Similarly, at 37°C, both the TS mutants expressing the N-terminal region (NTD) of *Pf*CENP-C show predominantly large bud morphology with fragmented nuclear mass along the mother-bud neck and a subset of unviable cells lacking detectable nuclear staining. These abnormal phenotypes reflect impaired chromosome segregation on MIF2p loss-of-function. At 37°C, the mif2-3 TS mutants expressing the *Pf*CENP-C motif show normal bud morphology but with chromosome mis-segregation (Figure 3E, black arrows) as cells show a single unsegregated G2 nucleus in the mother cell while the mif2-2 TS mutants expressing the *Pf*CENP-C motif show normal bud morphology and proper chromosome segregation. The majority of mif2-3 and mif2-2 TS mutant cells expressing *Pf*CENP-C dimerization domain (DD) show normal bud morphology with proper chromosome segregation apparent by the intracellular location of the nucleus that is equally segregated between the bud and the mother cell. The TS mutants expressing budding yeast MIF2p also show restoration of the normal phenotype at 37°C. The ability of *Pf*CENP-C motif in mif2-2 TS mutants and the *Pf*CENP-C dimerization domain in mif2-2 and mif2-3 TS mutants to rescue the mitotically arrested cells and overcome the chromosome segregation defects demonstrates their role in mediating accurate chromosome segregation. Collectively, these results suggest that the putative *Pf*CENP-C is a true CENP-C homologue.

### The dimerization domain of *Pf*CENP-C harbours functionally important F1993, F1996 and Y2069 residues

**Figure 4:**
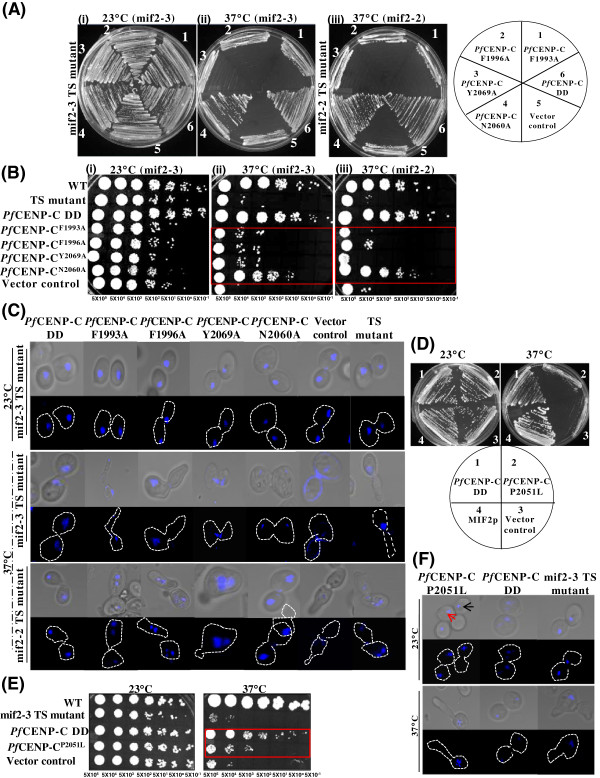
**Functionally essential residues lie within the** ***Pf*** **CENP-C dimerization domain.** (**A** and **B**, i-iii) Complementation and ten-fold serial dilution assays with the *Pf*CENP-C dimerization domain (DD) carrying mutations at the conserved residues essential for the CENP-C dimerization. (i) At 23°C, the different *Pf*CENP-C-DD mutants, *Pf*CENP-C^F1993A^, *Pf*CENP-C^F1996A^, *Pf*CENP-C^Y2069A^, *Pf*CENP-C^N2060A^ and vector control show normal growth. (ii and iii) At 37°C, both mif2-3 as well as mif2-2 TS mutants expressing the *Pf*CENP-C-DD mutants, *Pf*CENP-C^F1993A^, *Pf*CENP-C^F1996A^ and *Pf*CENP-C^Y2069A^ show complete growth arrest while the WT *Pf*CENP-C-DD and *Pf*CENP-C^N2060A^ show growth rescue. The red boxes show the comparative growth patterns of WT *Pf*CENP-C-DD and various *Pf*CENP-C-DD mutants in mif2-3 and mif2-2 TS mutants at 37°C. **(C)** Confocal images of various *Pf*CENP-C-DD mutants, *Pf*CENP-C^F1993A^, *Pf*CENP-C^F1996A^ and *Pf*CENP-C^Y2069A^ show severe phenotypes with deformed bud morphology and dispersed and /or fragmented nuclear staining in the TS mutants while the *Pf*CENP-C^N2060A^ show normal phenotype at 37°C. **(D and E)** The functional complementation assays with *Pf*CENP-C^P2051L^ corresponding to mif2-3 TS MIF2p^P505L^ mutation shows growth arrest at 37°C while the non-mutated WT *Pf*CENP-C-DD facilitates growth at 37°C in mif2-3 TS mutant cells (highlighted with red box). **(F)** The confocal images representing the gain-or loss-of-phenotypes of mif2-3 TS mutant cells expressing *Pf*CENP-C^P2051L^. At 23°C, *Pf*CENP-C^P2051L^ expressing TS mutants show both proper chromosome segregation (red arrows) as well as chromosome mis-segregation phenotype (black arrow) evident by the nucleus (blue) localization. At 37°C, *Pf*CENP-C^P2051L^ expressing TS mutants show abnormal phenotypes and most of the mif2-3 cells expressing *Pf*CENP-C^P2051L^ are unviable. The WT non-mutated *Pf*CENP-C-DD displays normal chromosome segregation phenotypes at both 23 and 37°C, respectively.

The dimerization domain of CENP-C is essential for centromere function, proper structuring of centromeric chromatin and *in vivo* kinetochore assembly [4, 6, 27, 29, 34]. To decipher the underlying residues essential for the functions of *Pf*CENP-C dimerization domain, point mutations in some of the conserved amino acid residues of this domain were incorporated. In budding yeast MIF2p, the residues Y451, F452 and F523 within the dimerization domain are shown to be essential for its dimerization ability upon which mutation results in arrested growth while the mutation of another conserved residue T488 which is present outside the dimer interface is dispensable for growth [6]. The multiple sequence alignment of the dimerization domain between *P. falciparum* and the budding yeast shows that the amino acid residues F1993, F1996 and Y2069 of *Pf*CENP-C dimerization domain corresponds to the residues Y451, F452 and F523 of MIF2p dimerization domain, respectively (Figure 1C, solid black arrows). As the *Pf*CENP-C dimerization domain contains an ‘isoleucine’ instead of ‘T488’ at the analogous position in MIF2p, another conserved residue, N2060 (Figure 1C, solid red arrow) in *Pf*CENP-C corresponding to N514 in MIF2p dimerization domain was mutated to ‘A’ to determine its role on growth and chromosome segregation. Since the functional dimerization domain is required for the normal growth in yeast [6], the functional complementation assays were performed to investigate the effect of *Pf*CENP-C dimerization domain mutants *Pf*CENP-C^F1993A^, *Pf*CENP-C^F1996A,^*Pf*CENP-C^Y2069A^ and *Pf*CENP-C^N2060A^ on the growth of TS mif2-3 and mif2-2 cells at the permissive (Figure 4A and B, i; see Additional file 2A and B) and non-permissive (Figure 4A and B, ii-iii) temperatures. The complementation (Figure 4A, i-iii; see Additional file 2A) and ten-fold serial dilution (Figure 4B, i-iii; see Additional file 2B) assays for monitoring the viability and phenotypes of mif2-3 and mif2-2 TS mutant cells, expressing the various *Pf*CENP-C dimerization domain mutants individually were tested. At the permissive temperature (Figure 4A and B ,i; see Additional file 2Aand B), the mif2-3 and mif2-2 TS cells expressing different *Pf*CENP-C dimerization domain show normal growth rate and colony size as the TS mutants expressing the WT *Pf*CENP-C dimerization domain (DD). At the non-permissive temperature (Figure 4A and B ,ii and iii), unlike the WT *Pf*CENP-C dimerization domain, the mif2-3 and mif2-2 TS mutant cells expressing *Pf*CENP-C^F1993A^, *Pf*CENP-C^F1996A^ and *Pf*CENP-C^Y2069A^ dimerization domains show significant growth reduction while both the TS mutants expressing *Pf*CENP-C^N2060A^ show unimpaired growth. The growth assays demonstrate the essentiality of the residues F1993, F1996 and Y2069 within the dimerization domain of *Pf*CENP-C while the residue N2060 is not essential for *Pf*CENP-C functions.

Further, the phenotypic changes exhibited by the dimerization domain mutants *Pf*CENP-C^F1993A^, *Pf*CENP-C^F1996A^, *Pf*CENP-C^Y2069A^ and *Pf*CENP-C^N2060A^ were assessed at 23 and 37°C, respectively (Figure 4C, see Additional file 2C). At 23°C, mif2-3 and mif2-2 TS mutant cells expressing different *Pf*CENP-C dimerization domains show proper chromosome segregation and normal bud morphology. At 37°C, ~90-94% of mif2-3 and mif2-2 TS mutant cells expressing *Pf*CENP-C^F1993A^, *Pf*CENP-C^F1996A^ and *Pf*CENP-C^Y2069A^ are mitotically arrested and unviable. This is evident by the aberrant bud morphologies with fragmented nucleus or loss of nuclear staining. These abnormal phenotypes are attributable to the failure of *Pf*CENP-C dimerization domain mutants to dimerize resulting in impaired growth, defective chromosome segregation and loss of viability. Both the TS mutants expressing *Pf*CENP-C^N2060A^ show normal bud morphology accompanied with proper chromosome segregation. The data presented here identify some of the key residues that are essential for the formation of a functional dimerization domain. This further demonstrates the role of the *Pf*CENP-C dimerization domain in mediating the *in vivo* CENP-C functions.

In *S. cerevisiae*, among the various mif2p TS mutants (chromosome segregation mutants), the mif2-3 TS mutant is very well characterized. The mif2-3 TS mutant has a point mutation, P505L in the MIF2p dimerization domain where proline at position 505 is substituted by leucine [34, 36]. At the non-permissive temperature, the MIF2p^P505L^ mutation confers the aberrant TS phenotypes to the mif2-3 TS mutants and renders the dimerization domain non-functional [6, 34, 36]. The amino acid residue proline is conserved in MIF2p, CENP-C and also in *P. falciparum* CENP-C at the analogous position (Figure 1C, broken black arrow). To confirm the complementation ability of *Pf*CENP-C dimerization domain, the conserved proline in the *Pf*CENP-C dimerization domain was substituted with leucine and the mutant *Pf*CENP-C^P2051L^ corresponding to the MIF2p^P505L^ mutation in the TS mif2-3 mutant was used for the studies. Figures 4D and E show the functional complementation and ten-fold serial dilution assays of mif2-3 TS mutants expressing *Pf*CENP-C^P2051L^ dimerization domain as compared to the mif2-3 TS mutants expressing the WT *Pf*CENP-C dimerization domain at permissive and non-permissive temperatures, respectively. At 23°C, TS mutants expressing the *Pf*CENP-C^P2051L^ dimerization domain display normal growth like the WT *Pf*CENP-C dimerization domain. However at 37°C, similar to the mif2-3 TS mutants and vector control, the TS mutants carrying the *Pf*CENP-C^P2051L^ dimerization domain too show complete growth arrest while normal growth is observed in the mif2-3 TS mutants expressing WT non-mutated *Pf*CENP-C dimerization domain. These results indicate that the *Pf*CENP-C^P2051L^ confers the TS phenotype.

The analysis was extended by examining the phenotypes exhibited by mif2-3 TS mutants expressing *Pf*CENP-C^P2051L^ dimerization domain both at permissive and non-permissive temperatures (Figure 4F). At 23°C, majority of mif2-3 TS mutants expressing *Pf*CENP-C^P2051L^ dimerization domain show normal bud morphology accompanied with chromosome mis-segregation phenotypes. Whereas at 37°C, similar to the untransformed mif2-3 TS mutant cells, the TS mutants expressing *Pf*CENP-C^P2051L^ dimerization domain show aberrant bud morphology, G2/M-arrest, elevated chromosome mis-segregation phenotypes, including unviable cells. The chromosome mis-segregation phenotype of TS mutants carrying the *Pf*CENP-C^P2051L^ dimerization domain even at the permissive temperature, (Figure 4F, black arrow) indicates that despite normal growth, the MIF2p functions are compromised. These chromosome segregation defects are further exacerbated at the non-permissive temperature, reflecting the loss of MIF2p function in the absence of a functional dimerization domain. The inability of the *Pf*CENP-C^P2051L^ dimerization domain to complement MIF2p can be attributed to the absence of a functional dimerization domain, which in turn fails to overcome the chromosome segregation defects in the mif2-3 TS mutants.

### *Pf*CENP-C dimerization domain is required for maintaining the mitotic spindle integrity

**Figure 5:**
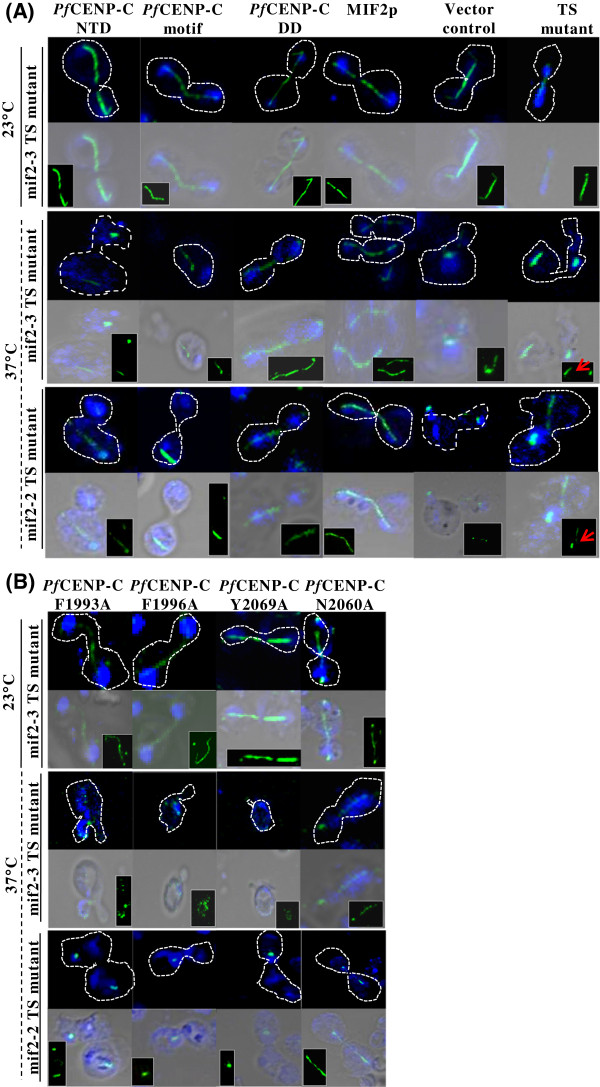
**The mitotic spindle integrity is maintained by the** ***Pf*** **CENP-C dimerization domain. (A)** Confocal images showing the mitotic spindle (green) and the Hoechst stained nucleus (blue) of the mif2-3 and mif2-2 TS mutant cells expressing the *Pf*CENP-C-NTD, -motif and -DD, region, MIF2p and empty vector alone at 23 and 37°C, respectively. At 23°C, all the mif2-3 TS mutants expressing different constructs show normal spindle structure connecting the nuclear masses across the bud and mother cell. At 37°C, both in mif2-3 and mif2-2 TS mutants except the *Pf*CENP-C-DD and MIF2p, all the other constructs show abnormal and discontinuous spindles. The spindle morphology (green) is also shown in the insets. The broken spindle in the mif2-3 and mif2-2 TS mutant cells are shown by red arrows in the insets. **(B)** The confocal images representing the disintegrated mitotic spindle morphologies in mif2-3 and mif2-2 TS mutant cells expressing the *Pf*CENP-C-DD mutants, *Pf*CENP-C^F1993A^, *Pf*CENP-C^F1996A^ and *Pf*CENP-C^Y2069A^ while *Pf*CENP-C^N2060A^ show normal mitotic spindles at 37°C. NTD- N-terminal region; DD-dimerization domain.

In *S. cerevisiae*, MIF2p is required for the maintenance of normal spindle structures. Fluorescence and electron microscopy in the large budded TS mif2-3 and mif2-2 cells have revealed the presence of short, discontinuous and broken spindles [6, 21]. To determine the spindle morphology in mif2-3 and mif2-2 TS mutant cells expressing various *Pf*CENP-C domains, indirect immunofluorescence assays by staining the transformed mif2-3 and mif2-2 TS strains with anti-tubulin antibodies were performed. The confocal images (Figure 5A, see Additional file 3A) show the mitotic spindle morphologies of both the TS mutants alone and the TS mutants expressing *Pf*CENP-C N-terminal region, *Pf*CENP-C motif, *Pf*CENP-C dimerization domain, and MIF2p individually at 23 and 37°C, respectively. At permissive temperature, continuous spindles with properly segregated nuclear masses across the budding cells are observed in the TS mutants expressing different *Pf*CENP-C domains. At 37°C, both the TS mutants expressing the *Pf*CENP-C N-terminal region and vector alone show discontinuous, unelongated and broken spindles as these cells with abnormal morphologies show no continuous tubulin staining along the nuclear mass, which is in concordance with the previous study of Brown *et al*. [21]. However, the mif2-3 and mif2-2 TS mutants expressing only the *Pf*CENP-C dimerization domain could rescue the abnormal spindle morphologies as continuous spindles connecting the two discrete nuclear masses in the budding cells are seen in ~ 65% and ~ 40% of the rescued cells, respectively. The mif2-3 and mif2-2 TS mutants expressing the *Pf*CENP-C motif show ~82% and ~68% cells with broken spindles, respectively. At 37°C, normal spindle morphologies are also observed in the TS mutants expressing yeast MIF2p. The aberrant spindle morphologies in the TS cells are apparent as compared to the TS cells rescued by *Pf*CENP-C dimerization domain. The presence of discontinuous spindles could be due either to the loss in connection between the spindles or an abrupt breaking of the spindles [21]. The discontinuous spindles might hamper the pole-ward movement of sister chromatids during anaphase ultimately contributing to the defective chromosome segregation [21]. The restoration of normal mitotic spindle structures on complementation with the dimerization domain of *Pf*CENP-C also demonstrates the *Pf*CENP-C dimerization domain-mediated proper chromosome segregation in the MIF2p deficient cells. The ability of dimerization domain to rescue the spindle defects in TS mutant cells raised the question whether the mitotic spindle integrity is disrupted on expression of *Pf*CENP-C dimerization domain mutants. The confocal images (Figure 5B, see Additional file 3B) show that at non-permissive temperature, none of the *Pf*CENP-C dimerization domain mutants (*Pf*CENP-C^F1993A^, *Pf*CENP-C^F1996A^ and *Pf*CENP-C^Y2069A^) could rescue the abnormal spindle morphology which is evident by the presence of disintegrated spindles in the mif2-3 and mif2-2 TS cells expressing all the above mentioned *Pf*CENP-C dimerization domain mutants. The mif2-3 and mif2-2 TS mutants expressing *Pf*CENP-C^N2060A^ show intact normal spindles confirming its dispensable role in chromosome segregation. The loss of spindle integrity in the absence of dimerization domain is consistent with a defective centromere protein. These results confirm the essential role of the *Pf*CENP-C dimerization domain in maintaining the mitotic spindles integrity during chromosome segregation.

### *Pf*CENP-C is enriched at *Plasmodium*centromeres and interacts with *Pf*CENH3 and α-tubulin *in vivo*

**Figure 6:**
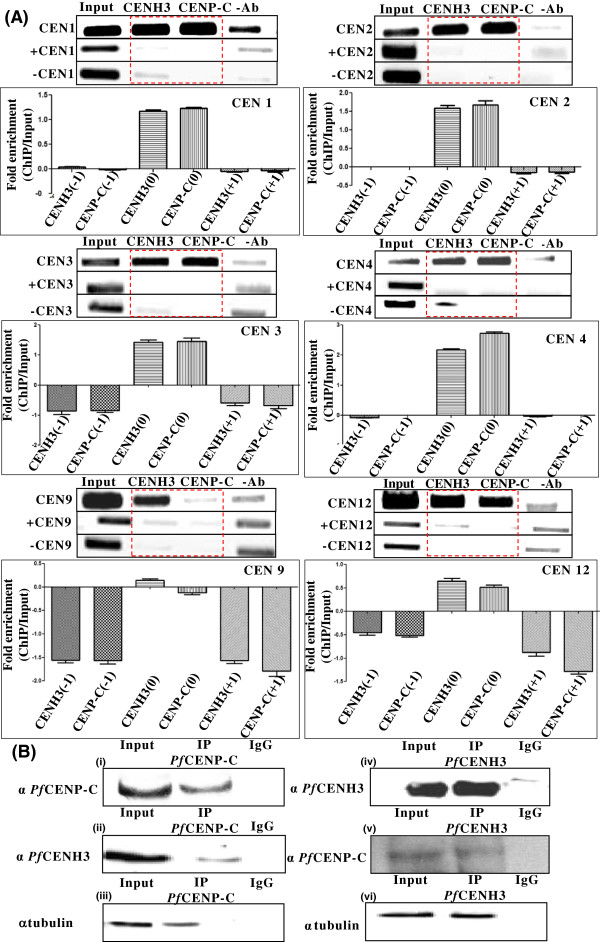
***In vivo*** **enrichment of** ***Pf*** **CENP-C on the** ***Plasmodium*** **centromeres and its** ***in vivo*** **interaction with** ***Pf*** **CENH3 and mitotic spindles. (A)** ChIP-PCR assay showing the enrichment of *Pf*CENP-C on the six *P. falciparum* centromeres (CEN 1, 2, 3, 4, 9, and 12) as compared to the non centromeric regions (red dotted boxes). +CEN and –CEN represents the non-centromeric regions lying upstream and downstream of each *P. falciparum* centromere, respectively. The non-centromeric regions show weak or no recruitment of *Pf*CENH3 and *Pf*CENP-C for all the six *P. falciparum* centromeres tested. The fold enrichment (ChIP over input) was determined for each centromere in comparison with the non-centromeric regions by quantifying the gel band intensities using AlphaImager. In the graphs (x-axis), CENH3 (+1), CENP-C (+1) and CENH3 (-1), CENP-C (-1) represents the ChIP with *Pf*CENH3 and *Pf*CENP-C at regions upstream and downstream to the centromeres, respectively. CENH3 (0) and CENP-C (0) in the x-axis of the graphs represent the *Pf*CENH3 and *Pf*CENP-C enriched centromeric regions, respectively. The y-axis represents the fold enrichment (ChIP over input) for each chromosome tested. **(B)** Immunoprecipitation assays with *Pf*CENP-C (panels, i-iii) and *Pf*CENH3 (panels, iv-vi) show the interaction of *Pf*CENP-C with *Pf*CENH3 (panel ii) and mitotic spindles (panel iii). *Pf*CENH3 also interacts with *Pf*CENP-C (panel v) and tubulin (panel vi).

To determine the enrichment of *Pf*CENP-C on centromeric regions *in vivo*, ChIP assays were performed as previously described [49, 50] with *Pf*CENP-C and *Pf*CENH3 antibodies (Figure 6A). As the centromeres are occupied by *Pf*CENH3 on all the *P. falciparum* centromeres [40], ChIP with anti-*Pf*CENH3 was used as a positive control. Semi-quantitative PCR analysis on *Pf*CENP-C and *Pf*CENH3 immunoprecipitated samples using the set of primers specific for the centromeric regions of six *P. falciparum* chromosomes (CEN 1, 2, 3, 4, 9, and 12) [40] were carried out. To validate the specificity of the assay, control primers from regions lying upstream (+CEN) and downstream (-CEN) of each centromere tested were designed (see Table 1 for the control primers) for the semi-quantitative PCR analyses on the ChIP samples followed by the quantification of the gel band intensities. The ChIP-PCR analyses show that like *Pf*CENH3, *Pf*CENP-C is also enriched on all the six *P. falciparum* centromeric regions analysed. However, at the non-centromeric sites, upstream as well as downstream of each centromere, very weak or no enrichment of *Pf*CENH3 and *Pf*CENP-C is observed. The *in vivo* enrichment of *Pf*CENP-C on the centromeric regions confirms its functions as a centromere protein in *P. falciparum*. Like *Pf*CENH3, the results also establish *Pf*CENP-C as the centromere binding protein in the parasite.

**Table 1:** **Set of primers for amplification of** ***P. falciparum*** **non-centromeric regions**

Primer name	Nucleotide sequence (5′ to 3′)
NONCEN 1-FP	CATCACCTGTCATAGCAACTG
NONCEN 1-RP	GTTGTTATAATGGAAGAGAATTTGAAG
NONCEN 1-FP	GTAAAAGGAAAGAAGAAAAGAGTAGAG
NONCEN 1-RP	ACAATCATTAGTAACATATTCACGAAC
NON-CEN 2-FP	GATAAAAATGTTATATTAGGTTCAAAAG
NON-CEN 2-RP	GGAATATGGTATCTCTATCTATAG
NON-CEN 2-FP	ACCCAAGTTAGTTAAGTTAAGACC
NON-CEN 2-RP	TTCACTAACATAGGTCTTACATTC
NON-CEN 3-FP	ACCTATGTTAGTATCTACATGACC
NON-CEN 3-RP	AGGTCTTACTCTCACTGATATAG
NONCEN 3-FP	TACATCATACCCACCGTCCAATC
NONCEN 3-RP	GATTTCAAGTCATTATTTTGTTTGTACGAG
NONCEN 4-FP	ATTTATTATTTCCCCTTTCATCATTTTTATG
NONCEN 4-RP	ATAGCGGAAGTCCTAGAACATC
NONCEN 4-FP	AAAATATGTAGAGGACTATGAATCAGATG
NONCEN 4-RP	AATAAATGATTATCCTCTACATCTTGATTC
NONCEN 9-FP	GCAAATACTAAATGTGTTCGTATGGG
NONCEN 9-RP	TATTTGGATCAATGTACCGAATTTACTC
NONCEN 9-FP	TATATTCTAAGAAAAATCTGAAATCCTTC
NONCEN 9-RP	GACTAAGGATATGTGGAATCGTG
NONCEN 12-FP	GACATTCAGGGGATCATTATTACATTC
NONCEN 12-RP	TGATTGCTTTACTATAAATAATAAGAATGAAG
NONCEN 12-FP	CTTTCAAATAATTTTATATCTACATCCGTTTTTG
NONCEN12-RP	GAAAAATGTGATGAGAAAGTAGGAAGTC

The set of primers used for the amplification of non-centromeric regions upstream and downstream of the *P. falciparum* centromeres. NONCEN refers to non-centromeric regions and the numbers represent the chromosome numbers.

The co-occupancy of *Pf*CENH3 and *Pf*CENP-C on the centromeres and their co-localization throughout the cell cycle [39] prompted to check the *in vivo* interactions of *Pf*CENH3with *Pf*CENP-C by co-immunoprecipitation (co-IP) assay. The pull-down of *Pf*CENH3 using anti-*Pf*CENP-C antibodies and *vice-versa* were performed. The presence of *Pf*CENH3 and *Pf*CENP-C in the pull-down immunocomplexes was confirmed by the Western blot analyses (Figure 6B, panels i-vi). The co-IP assays with anti-*Pf*CENP-C show its association with *Pf*CENH3 and α-tubulin, marker for mitotic spindles (Figure 6B, panels ii and iii). Similarly, co-IP assays using anti-*Pf*CENH3 could pull down both *Pf*CENP-C and α -tubulin (Figure 6B, panels v and vi). The association of *Pf*CENP-C with *Pf*CENH3 and mitotic spindles suggests the conserved mechanism of centromere-kinetochore assembly in *P. falciparum*.

## Discussion

*Pf*CENP-C is constitutively expressed throughout the *Plasmodium* cell cycle and like *Pf*CENH3, localizes to the centromeres at the perinuclear regions. Employing functional complementation of MIF2p by *Pf*CENP-C domains, this study reports the functions of *Pf*CENP-C, emphatically its dimerization domain to understand its role in chromosome segregation and mitotic events. The results establish *Pf*CENP-C as a true homologue of CENP-C. Thus, this study delineates the functions of the dimerization domain of *Pf*CENP-C in *P. falciparum*.

In yeast and humans, the dimerization domain of CENP-C is reported to be essential for growth, self association and kinetochore assembly. The ability of *Pf*CENP-C motif and the dimerization domain to rescue the loss of function in the TS MIF2p mutant cells is evident by the restoration of normal phenotype and proper chromosome segregation. The results indicate that the *Pf*CENP-C dimerization domain is more efficient than the *Pf*CENP-C motif in overcoming the MIF2p loss-of-function. The inability of the C-terminal deletion mutants of *Pf*CENP-C to overcome the growth and chromosome segregation defects indicate that the N-terminal region of *Pf*CENP-C is dispensable for the growth but the possibility of this region having other functions cannot be ruled out and needs experimental validation. The possibility of interaction of endogenous N-terminal region of MIF2p with *Pf*CENP-C motif and dimerization domain resulting in a fully functional protein that mediates the proper chromosome segregation also cannot be ruled out.

CENP-C when expressed at physiological level localizes exclusively to the centromeres but on overexpression, displays mistargeting to the non-centromeric chromatin regions [53]. The overexpressed CENP-C in chicken DT40 cells localizes to both the centromere as well as the chromosomal arms indicating its mislocalization [24]. The mislocalization of *Pf*CENP-C motif and *Pf*CENP-C dimerization domain in the transformants might be the consequence of its overexpression under the strong ADH promoter in the p415ADH vector, which prevents the specific centromeric localization of these domains. Alternatively, as the N-terminal domain of CENP-C regulates the CENP-C expression by destabilizing the excess CENP-C, an inherent mechanism to ensure centromere-specific localization of CENP-C [30] and as both the *Pf*CENP-C motif and *Pf*CENP-C dimerization domain constructs lack the N-terminal region of *Pf*CENP-C, suggests the possibility that the mislocalization of the *Pf*CENP-C domains might be due to the absence of destabilizing function performed by its N-terminal region.

In budding yeast, the mutations in the conserved residues Y451S, F452A and F523A within the MIF2p dimerization domain have been shown to break the contacts between the dimer, possibly by disrupting the conformation of strand connecting the two β-sheets of MIF2p, which destabilizes the dimerization domain [6]. The studies presented here indicate that the corresponding *Pf*CENP-C residues F1993, F1996 and Y2069, respectively, too are essential for the stable and functional dimerization domain unlike the conserved *Pf*CENP-C N2060 residue, which is dispensable. The TS mutant mif2-3 harbours a MIF2p^P505L^ mutation in its dimerization domain that is responsible for the TS characteristics and the aberrant phenotypes exhibited by the mif2-3 TS mutants at the non-permissive temperature [6, 34, 36]. The inability of the *Pf*CENP-C^P2051L^ dimerization domain mutant corresponding to MIF2p^P505L^ mutation to rescue the growth and phenotypic defects in the mif2-3 TS mutants at the non-permissive temperature lends direct evidence for *Pf*CENP-C functional dimerization domain in mediating its functions.

Thus, the disruption of the *Pf*CENP-C dimerization domain results in the abolishment of kinetochore assembly and mitosis in yeast, confirmed by its detrimental effects on the mitotic spindles, chromosome segregation and the cell viability, providing evidence that the mutations in the conserved residues of *Pf*CENP-C dimerization domain might also disrupt its dimer formation which in turn affects its high-order chromatin organization at the centromeres and renders the chromosome segregation machinery impaired.

It has been proposed that the loss of spindle integrity in the MIF2p-deficient cells is due to the defective spindle elongation during the anaphase but not the failure in spindle formation [34]. CENP-C has also been shown to maintain the kinetochore size as the impaired CENP-C functions lead to reduced diameter of kinetochores that may weaken or loosen the binding to the mitotic spindles [13]. Borrowing these concepts from the studies of Brown *et al.* and Tomkiel *et al.,* the plausible explanation for the aberrant and disintegrated mitotic spindles in the mif2-3 and mif2-2 cells expressing the *Pf*CENP-C N-terminal region, *Pf*CENP-C motif and the *Pf*CENP-C dimerization domain mutants (*Pf*CENP-C^F1993A^, *Pf*CENP-C^F1996A^ and *Pf*CENP-C^Y2069A^) may be due to the failure of *Pf*CENP-C to recruit and interact with the proteins that generate the stable spindle tension during the sister chromatids separation. This imbalance in the tension thus results in the abrupt breakage of the mitotic spindles. Alternatively, the spindles may fall apart due to the impaired spindle elongation during anaphase, resulting in structurally defective spindles. However, the spindle defects in the TS mutants are overcome by the *Pf*CENP-C dimerization domain demonstrating its role in the maintenance of the structure of mitotic spindles. This also supports the idea that the *Pf*CENP-C dimerization domain plays an important role in the kinetochore assembly at the centromeres and establishes connection with the mitotic spindles to ensure accurate chromosome segregation. The enrichment of *Pf*CENP-C on the centromeric regions of *P. falciparum* as compared to the non-centromeric regions and its interaction with *Pf*CENH3 and microtubules further strengthens its centromeric function in the malaria parasite.

These findings thus offer insights into the structural-functional details of various *Pf*CENP-C domains especially the dimerization domain and its role in formation of a functional centromere-kinetochore structure.

## Conclusions

The two conserved domains of *Pf*CENP-C, the *Pf*CENP-C motif and the *Pf*CENP-C dimerization domain have been mapped by bio-informatic analysis. The functional complementation assays establish the putative *Pf*CENP-C as a true CENP-C homologue involved in chromosome segregation, stable mitotic spindle morphology and cell viability. The key residues within the *Pf*CENP-C dimerization domain essential for its *in vivo* functions as a centromere protein have also been identified. The binding of *Pf*CENP-C to the *P. falciparum* centromeres and its *in vivo* interactions with *Pf*CENH3 and mitotic spindles further strengthens its centromere-associated functions.

## Electronic supplementary material

Additional file 1: **Functional complementation of** ***Saccharomyces cerevisiae*** **MIF2p (A and B).** Complementation and ten-fold serial dilution assays with mif2-2 TS mutant expressing various *Pf*CENP-C domains: -NTD, -motif and -DD, MIF2p and vector control at 23°C. **(C)** (i) The confocal images show the normal phenotypes of mif2-2 TS mutants carrying various *Pf*CENP-C domains, MIF2p, vector control and the parent strain alone at 23°C. The nucleus (blue) is stained with Hoechst. (ii and iii) The graphs show the percentage of normal phenotype (y-axis) exhibited by mif2-3 and mif2-2 TS mutant cells expressing various *Pf*CENP-C domains, MIF2p, vector control and the parent TS mutant strain (x-axis) at 37°C. The normal phenotypes were scored for the cells showing both the normal bud morphology as well as proper chromosome segregation. Number of cells counted = 100. NTD- N-terminal region, DD-dimerization domain. (PDF 140 KB)

Additional file 2: **Functionally essential residues within the** ***Pf*** **CENP-C dimerization domain. (A and B)** Growth and ten-fold serial dilution assays with the mif2-2 TS mutants expressing the *Pf*CENP-C-DD mutants, *Pf*CENP-C^F1993A^, *Pf*CENP-C^F1996A^, *Pf*CENP-C^Y2069A^ and *Pf*CENP-C^N2060A^ at 23°C. All the constructs show normal growth at permissive temperature. **(C)** The phenotypes of mif2-2 TS mutants expressing *Pf*CENP-C^F1993A^, *Pf*CENP-C^F1996A^, *Pf*CENP-C^Y2069A^ and *Pf*CENP-C^N2060A^ at 23°C. The Hoechst stained nucleus (blue) shows proper chromosome segregation in all the constructs. (PDF 86 KB)

Additional file 3: **The mitotic spindle integrity is maintained by the dimerization domain. (A)** Confocal images showing the mitotic spindle (green) and the Hoechst stained nucleus (blue) of the mif2-2 TS mutant cells expressing the *Pf*CENP-C domains: -NTD, -motif and -DD, MIF2p and empty vector alone at 23°C. **(B)** The confocal images represent the mitotic spindles of mif2-2 TS mutants expressing *Pf*CENP-C-DD mutants, *Pf*CENP-C^F1993A^, *Pf*CENP-C^F1996A^, *Pf*CENP-C^Y2069A^ and *Pf*CENP-C^N2060A^ at 23°C. At permissive temperature, normal spindle morphology is observed by all the constructs expressed by mif2-2 TS mutants. The insets show the mitotic spindle structures. (PDF 218 KB)

## Abbreviations

TS: Temperature sensitive
WT: Wild type
NTD: N-terminal region
DD: Dimerization domain.
