# Supplementary figures and images for: The dimerization domain of PfCENP-C is required for its functions as a centromere protein in human malaria parasite Plasmodium falciparum
Source: Malar J. 2014 Dec 4;13:475. doi: 10.1186/1475-2875-13-475 (PMC4295259; doi:10.1186/1475-2875-13-475)

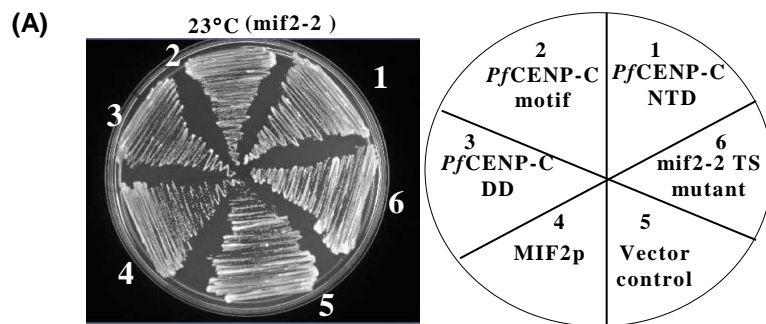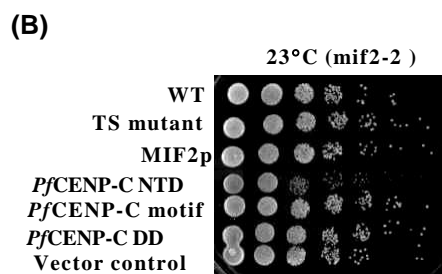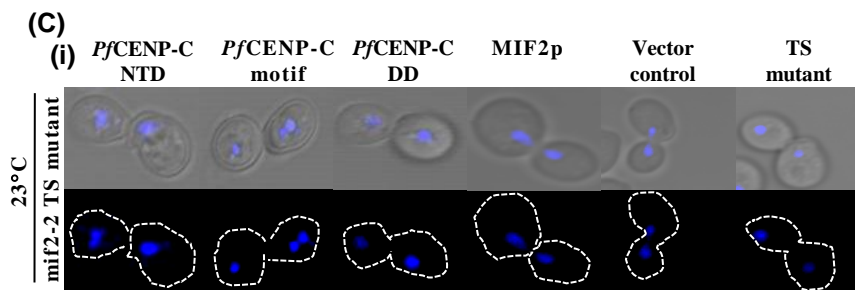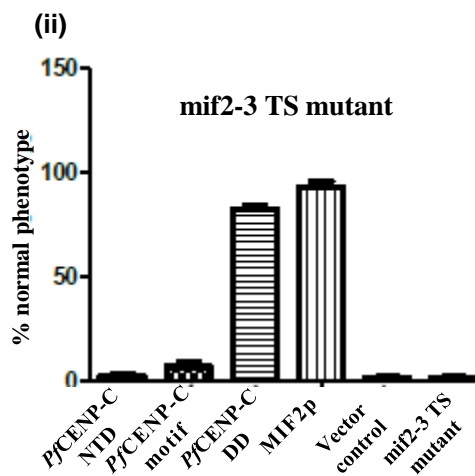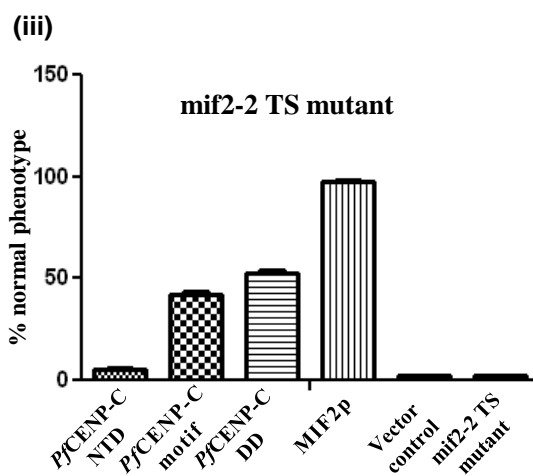

Supplement: Supplementary file 1 — Additional file 1: Functional complementation of Saccharomyces cerevisiae MIF2p (A and B). Complementation and ten-fold serial dilution assays with mif2-2 TS mutant expressing various PfCENP-C domains: -NTD, -motif and -DD, MIF2p and vector control at 23°C. (C) (i) The confocal images show the normal phenotypes of mif2-2 TS mutants carrying various PfCENP-C domains, MIF2p, vector control and the parent strain alone at 23°C. The nucleus (blue) is stained with Hoechst. (ii and iii) The graphs show the percentage of normal phenotype (y-axis) exhibited by mif2-3 and mif2-2 TS mutant cells expressing various PfCENP-C domains, MIF2p, vector control and the parent TS mutant strain (x-axis) at 37°C. The normal phenotypes were scored for the cells showing both the normal bud morphology as well as proper chromosome segregation. Number of cells counted = 100. NTD- N-terminal region, DD-dimerization domain. (PDF 140 KB) [file 12936_2014_3676_MOESM1_ESM.pdf]

(A)

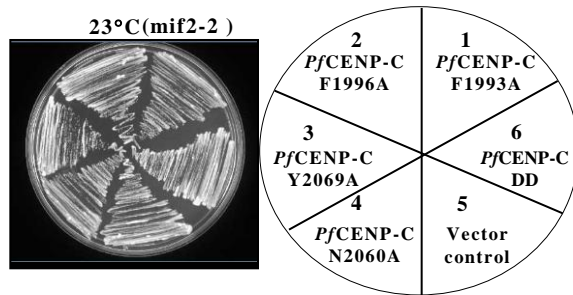

(B)

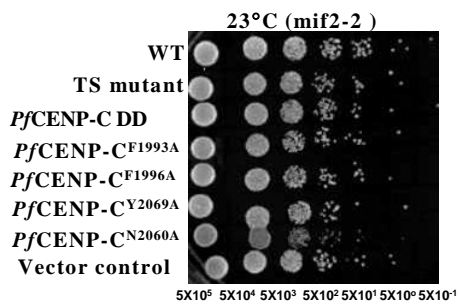

(C)

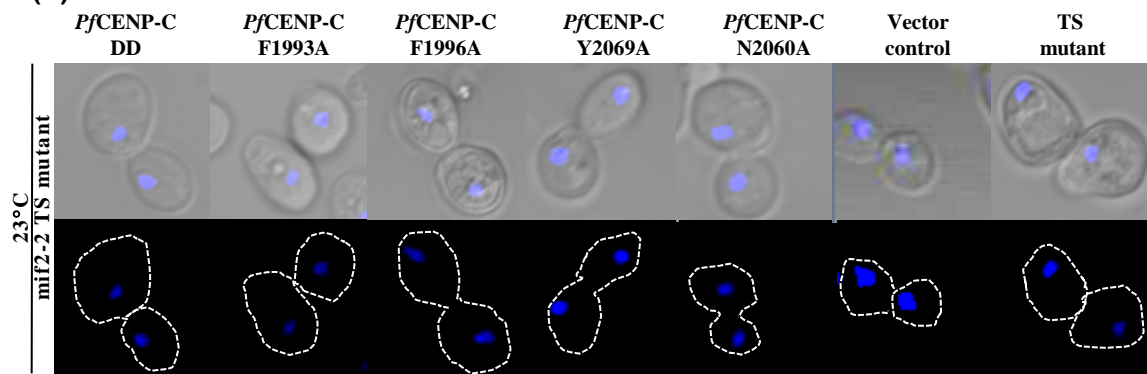

Supplement: Supplementary file 2 — Additional file 2: Functionally essential residues within the Pf CENP-C dimerization domain. (A and B) Growth and ten-fold serial dilution assays with the mif2-2 TS mutants expressing the PfCENP-C-DD mutants, PfCENP-CF1993A, PfCENP-CF1996A, PfCENP-CY2069A and PfCENP-CN2060A at 23°C. All the constructs show normal growth at permissive temperature. (C) The phenotypes of mif2-2 TS mutants expressing PfCENP-CF1993A, PfCENP-CF1996A, PfCENP-CY2069A and PfCENP-CN2060A at 23°C. The Hoechst stained nucleus (blue) shows proper chromosome segregation in all the constructs. (PDF 86 KB) [file 12936_2014_3676_MOESM2_ESM.pdf]

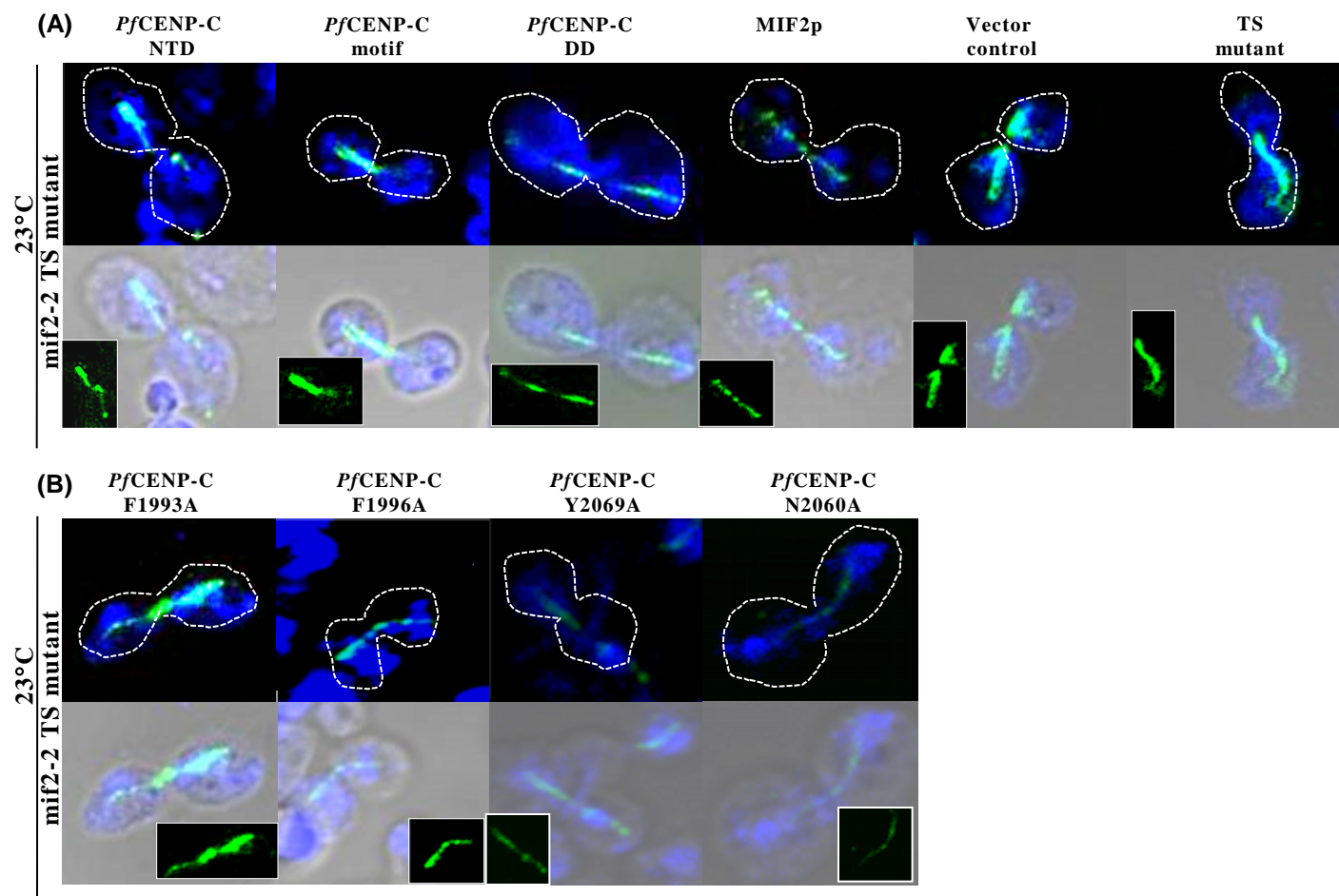

Additional file 3

Supplement: Supplementary file 3 — Additional file 3: The mitotic spindle integrity is maintained by the dimerization domain. (A) Confocal images showing the mitotic spindle (green) and the Hoechst stained nucleus (blue) of the mif2-2 TS mutant cells expressing the PfCENP-C domains: -NTD, -motif and -DD, MIF2p and empty vector alone at 23°C. (B) The confocal images represent the mitotic spindles of mif2-2 TS mutants expressing PfCENP-C-DD mutants, PfCENP-CF1993A, PfCENP-CF1996A, PfCENP-CY2069A and PfCENP-CN2060A at 23°C. At permissive temperature, normal spindle morphology is observed by all the constructs expressed by mif2-2 TS mutants. The insets show the mitotic spindle structures. (PDF 218 KB) [file 12936_2014_3676_MOESM3_ESM.pdf]
